# Short-term follow-up of masticatory adaptation after rehabilitation with an immediately loaded implant-supported prosthesis: a pilot assessment

**DOI:** 10.1186/s40729-017-0070-x

**Published:** 2017-03-07

**Authors:** Mihoko Tanaka, Collaert Bruno, Reinhilde Jacobs, Tetsurou Torisu, Hiroshi Murata

**Affiliations:** 1 Department of Prosthetic Dentistry, Graduate School of Biomedical Science, Nagasaki University, 1-7-1 Sakamoto, Nagasaki, 852-8588 Japan; 2 Centre for Periodontology and Implantology Leuven, IJzerenmolenstraat 110, B-3001 Heverlee, Belgium; 3 OMFS IMPATH, Department of Imaging & Pathology, University of Leuven, Kapucijnenvoer 33, BE-3000 Leuven, Belgium; 4 Oral and Maxillofacial Surgery, University Hospitals Leuven, Kapucijnenvoer 33, BE-3000 Leuven, Belgium

**Keywords:** Physiologic adaptation, Masticatory function, Immediate loading, Dental implants

## Abstract

**Background:**

When teeth are extracted, sensory function is decreased by a loss of periodontal ligament receptions. When replacing teeth by oral implants, one hopes to restore the sensory feedback pathway as such to allow for physiological implant integration and optimized oral function with implant-supported prostheses. What remains to be investigated is how to adapt to different oral rehabilitations.

The purpose of this pilot study was to assess four aspects of masticatory adaptation after rehabilitation with an immediately loaded implant-supported prosthesis and to observe how each aspect will recover respectively.

**Methods:**

Eight participants with complete dentures were enrolled. They received an implant-supported acrylic resin provisional bridge, 1 day after implant surgery. Masticatory adaptation was examined by assessing occlusal contact, approximate maximum bite force, masticatory efficiency of gum-like specimens, and food hardness perception.

**Results:**

Occlusal contact and approximate maximum bite force were significantly increased 3 months after implant rehabilitation, with the bite force gradually building up to a 72% increase compared to baseline. Masticatory efficiency increased by 46% immediately after surgery, stabilizing at around 40% 3 months after implant rehabilitation. Hardness perception also improved, with a reduction of the error rate by 16% over time.

**Conclusions:**

This assessment demonstrated masticatory adaptation immediately after implant rehabilitation with improvements noted up to 3 months after surgery and rehabilitation. It was also observed that, despite gradually improved bite force in all patients, masticatory efficiency and food hardness perception did not necessarily follow this tendency. The findings in this pilot may also be used to assess adaptation of oral function after implant rehabilitation by studying the combined outcome of four tests (occlusal contact, maximum bite force, masticatory efficiency, and food hardness perception).

## Background

Tooth loss represents a major oral disability comparable to an amputation, with severe impairment of oral functions [1]. While denture wearers can rely on mucosal sensors, anchoring prosthetic teeth to the bone via osseointegrated implants has been assumed to create a (partial) sensory substitution for missing periodontal ligament receptors from stimuli transmitted via the bone [2]. The restoration of the sensory feedback pathway is necessary for the physiological integration of implant-supported prostheses in the human body. It helps to optimize essential oral functions, such as chewing and biting. Studies on such functions usually report an improvement of oral functions with implant-supported prostheses as opposed to conventional dentures [3–9]. Improved oral function also impacts on quality of life [10], often scored with ratings for function, pain, discomfort, and psychosocial factors using the GOHAI system [11]. However, one should realize that such rehabilitation may also create some patient-related masticatory and other problems or complications [12]. Such complaints could be related to uncomfortable occlusion, accidental biting of the cheek or tongue, or problems during speech. Other complications might include fractures of prosthetic or implant components. For adequate mastication, the ability to adapt to food of various levels of hardness and various volumes is important. In individuals with natural dentition, such information is processed by the periodontal ligament receptors [13–15]. Since patients with implant-supported prostheses lose the periodontal ligament and its elaborate associated peripheral feedback mechanism, it is possible that they are not able to differentiate food hardness and texture. In this context, it is important to mention that some studies reported no significant improvement of masticatory function after implant treatment [3, 16, 17]. Jacobs et al. [3] indeed noticed that some of these patients might realize that the peripheral feedback mechanism is no longer assisting them, rendering some of them afraid of biting too hard. [3] Instead, these anxious patients are found to bite submaximally with implant-supported prostheses [3].

In addition, it also remains to be demonstrated how a potential compensatory mechanism might work, with one of the options being osseoperception [2, 18–23]. In this context, it is also important to consider the adaptation time needed after oral rehabilitation. Some studies have performed longitudinal evaluations of masticatory function for more than 3 years [24, 25]. However, there are limited data available on short-term adaptation to mastication, especially in the first months after being fitted with a prosthetic appliance. Although approximately 2 months are generally required for adaptation to a new removable denture, the time needed to adapt to a new implant-supported prosthesis has not been established [26]. Furthermore, adaptation is likely to be more difficult with full fixed implant prostheses [27].

In a functional magnetic resolution imaging (fMRI) study of patients with implants, it was demonstrated that punctate mechanical stimulation of oral implants activates both primary and secondary cortical somatosensory areas and was suggested that brain plasticity occurs when extracted teeth are replaced by endosseous implants [28]. In another fMRI study, it was suggested that the time after tooth extraction may affect neural plasticity, which in turn can influence osseoperception, with the amount of time possibly being an indicator for prosthetic treatment planning [23]. The lack of peripheral feedback mechanisms in patients with implant-supported full fixed prostheses may lead to a lack of control over the biting force [3, 29]. Such control is needed for refinement and control of the biting force for various types of food [7, 30–32]. While patients with implant-supported bridges are able to bite food with varying levels of hardness, it could be questioned whether they are able to differentiate between the hardness variations and thus apply an adapted chewing pattern [33]. Although some studies have demonstrated the tactile function of patients with oral implants [18, 19], the perception of food hardness is yet another sensory function that should be evaluated in order to obtain more information on modulation and masticatory adaptation. However, there have been few studies on this issue. Although adaptation to food texture during mastication by dentate subjects has been tested [34], it has not yet been followed up in patients receiving implant placement. In a recent cross-sectional study, mastication adaptability in patients with implant-supported bridges was assessed with soft and hard food models using an electromyogram (EMG) [7]. Patients with implants showed a significantly weaker increase in EMG activity with increased food hardness. In addition, muscular work performance (bite-force ratio and muscle activity) was found to be lower in patients with implants [35]. Furthermore, less coordinated masticatory muscle activity was found in patients with implant-supported prostheses [36].

The purpose of this pilot investigation was to use testing methodologies involving four aspects of masticatory adaptation after rehabilitation with an immediately loaded implant-supported prosthesis and to observe the recovery of each aspect respectively. Our hypothesis is that bite force may recover quickly, but other aspects will require monitoring and recording in order to form an overall judgment on the oral adaption to implant rehabilitation.

## Methods

Six females and 2 males (average age 66.4 years, range 52–85 years) with upper (*n* = 7) or lower (*n* = 1) complete dentures participated in this study. Inclusion criteria were (1) an opposite jaw that included natural dentition at least to the second premolar on both sides, (2) a need for fixed rehabilitation, (3) no medical contraindication to the placement of implants, (4) no need for augmentation procedures, and (5) willingness to participate in this study. The only exclusion criterion was temporo-mandibular dysfunction, since it may interfere with chewing and biting patterns and abilities. In the mandible 5 and in the maxilla, 6 OsseoSpeed implants and Uni Abutments 20° (Astra Tech, Mölndal, Sweden) were used to provide support for fixed rehabilitation. All participants were treated at the Center for Periodontology and Implantology, Leuven, Belgium, by the same surgeon (BC). Informed written consent with regard to treatment and masticatory function and follow-up procedures was provided to each participant. The study was approved by the ethics committee of the Catholic University of Leuven (B322201319432).

The day after implant surgery, implants were loaded with screw-retained implant-supported acrylic resin provisional restoration (immediate loading) as previously described [37, 38]. All provisional bridges extended to the second premolar or first molar region.

### Occlusal contact area and approximate maximum bite force measurements

Patient’s head was positioned with the Frankfort plane parallel to the floor. After opening the mouth, a pressure-sensitive sheet (Dental Prescale, 50H, type R, 97 μm thick, GC, Tokyo, Japan) was inserted on the occlusal plane. Patients were instructed to bite onto the test sheet as hard as possible for 3 s in the intercuspal position. This was repeated three times in each patient. The sheets were analyzed using special analytical equipment (Occluzer FPD-707, GC, Tokyo, Japan), namely, an analyzing device that could calculate bite force (N) and occlusal contact area (mm^2^) from the degree of discoloration of the pressure-sensitive sheets. Values from three sheets were averaged for each measurement, as described in a previous study [39]. In a pilot study, dentate patients (*n* = 14, mean age 58.4 ± 12.6 years) showed an occlusal contact area of 20.79 ± 8.10 mm^2^ and a maximal bite force of 696.8 ± 237.5 N.

### Measurement of masticatory efficiency

**Fig. 1:**
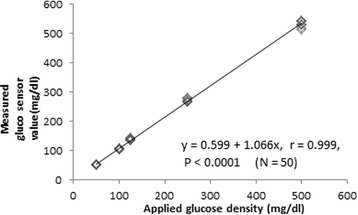
Correlation between measured Glucosensor value (mg/dl) (the *vertical axis*) and applied glucose density (mg/dl) (the *horizontal axis*) in the in vitro setup. A linear regression line could be applied to the data set, and we tested the accuracy of Glucosensor value

To assess the masticatory efficiency, we used glucose extraction in the filtrate obtained after chewing the specimen. After rinsing the mouth with tap water, a gum-like specimen mixed with 5% glucose with a height of 10 mm (Glucosensor Gummy, GC, Tokyo, Japan) was placed on patient’s tongue with chopsticks. Patients were requested to chew on the cube for 20 s, after which, they expectorated all the chunks of the cube into a cup equipped with a mesh filter to hold the debris. Thereafter, they rinsed their mouth again with 10 ml of water and expectorated into the same cup. The amount of glucose extraction in the filtrate obtained after chewing the specimen was used as a measure of masticatory efficiency. Glucose concentration in the filtrate (mg/dl) was measured using a calibrated Glucose Sensor Set (Glucosensor GS-1, GC, Tokyo, Japan), which utilizes a glucose sensor for diabetics (Accu-check Comfort, Roche Diagnostic, Basel, Switzerland) to measure masticatory efficiency according to a previous study, which reported its reliability for the evaluation of masticatory function [40]. For reproducibility, we tested the glucose concentration of control glucose solutions (500, 250, 125, 100, and 50 mg/dl) with the glucose sensor. The linear relationship that was observed between the glucose density of the solution (*x*) and the masticatory efficiency (the value of the glucose sensor) (*y*) is displayed in a scatter diagram (Fig. 1). The linear regression equation and Pearson’s correlation coefficient were as follows: *y* = 0.599 + 1.066*x*, *r* = 0.99 (*n* = 50, *p*< 0.0001). The intra-class correlation coefficient (ICC) is a prominent statistic to measure the test-retest reliability of data. The ICC (1, 3) of the data by Glucosensor was *p* = 1.000 (*n* = 5).

### Food hardness assessment

Three types of chewing specimen with different levels of hardness (hard, medium, and soft), with the same size and taste, were produced from sucrose (800 g), glucose (870 g), sorbitol (1000 g), gelatin (hard, 390 g; medium, 240 g; and soft, 150 g), Arabia gum (hard, 36 g), citric acid (42 g), lemon juice (15 g), and water and were 15 × 15 ×10 mm in size. The hardness of each type was determined under maximal stress during compression of 9 mm with a crosshead speed of 100 mm/min with a tooth-shaped jig using a texture analyzer (EZ test, Shimadzu Co., Kyoto, Japan). The hardness results were 73 ± 1.5 N for the soft, 88 ± 1.5 N for the medium, and 171 ± 1.9 N for the hard specimens.

To assess the hardness differences, the examiner placed each test specimen on the tongue with chopsticks, and then the participants chewed on all sides and swallowed. They were asked to remember the hardness of the first specimen, which always had medium hardness and served as a control, and then to determine the level of hardness (hard, medium, or soft) of four consecutive and randomly administered specimens by comparing them with the first one. This test was conducted in a double-blind manner to eliminate examiner bias.

The number of correct answers of hardness was used as a measure of hardness recognition. The subjects were allowed to expectorate any specimen that could not be chewed well enough to be swallowed and could change their answers until the last specimen was chewed.

### Data collection

Occlusal contact area, maximum bite force measurements, masticatory efficiency, and discriminating hardness assessments were performed on four occasions: (1) before implant surgery with the complete denture in situ, (2) 3 h after surgery, (3) 1–2 weeks, and (4) 3 months after insertion of the provisional screw-retained restoration.

### Statistical analysis

Considering the small sample size in the present psychophysical experiments, the option was taken to report mainly the descriptive statistics, in terms of average (SD, range) values for bite force, occlusal contact area, glucose concentration, and number of correct answers regarding hardness. Some nonparametric analyses were added in the difference between baseline prior to surgery and the follow-up data (Wilcoxon test, SPSS for Macintosh ver.21, SPSS, Chicago, USA). A *p* value <0.05 was considered to be statistically significant.

## Results

Two participants were unavailable to attend the testing at 1–2 weeks after the provisional restoration had been inserted, which resulted in missing data.

Overall descriptive analyses yielded the following observations for the four tests.

### Occlusal contact area and maximum bite force

**Fig. 2:**
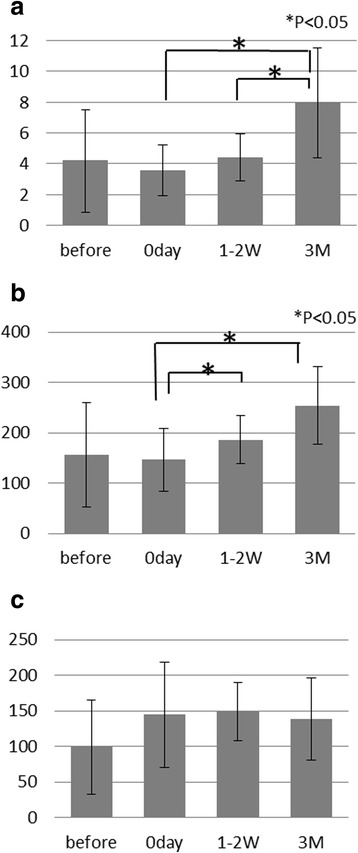
**a** Mean and standard deviation (SD) of occlusal contact area at each of the four times. The *horizontal label axis* was the time stage (1) before implant surgery with the complete denture in situ and (2) right after with provisional implant, (3) 1–2 weeks and (4) 3 months after insertion of the provisional screw-retained restoration, and the label to the *vertical axis* was contact area (mm^2^). The occlusal contact area was increased at 3 months after wearing implants (paired *t* test, *p* < 0.005). *p < 0.005, significant difference between conditions. **b** Mean and standard deviation (SD) of bite force at each of the four times. The *horizontal label axis* was the time stage, and the label to the *vertical axis* was bite force (N). The approximate maximum bite force was increased at 3 months after wearing implants (paired *t* test, *p* < 0.005). *p < 0.005, significant difference between conditions. **c** Mean and standard deviation (SD) of glucose data at each of the four times. The *horizontal label axis* was the time stage, and the label to the vertical axis was glucose data of Glucosensor value (mg/dl)

Occlusal contact and approximate maximum bite force were significantly increased 3 months after implant rehabilitation because of the adjustment of provisional occlusion, with the bite force gradually building up by 72% compared with that at stage one (prior to implant rehabilitation). Prior to implant surgery, when participants were wearing complete dentures for the lower or upper jaw, none expressed satisfaction with their dentures when we asked about them. However, occlusal contact and approximate maximum bite force varied widely among subjects but steadily increased in the individual participants (bite force, range 16.4–339.80 N, SD = 103.89; occlusal contact area, 0.4–9.63 mm^2^, SD = 3.31). The occlusal contact area was increased right after implant surgery (*p* < 0.005) and 3 months after wearing implants (*p* < 0.005). At the same time, maximum bite force also increased on these occasions (*p* < 0.001 and *p* < 0.005) (Fig. 2a, b). There was a positive and significant correlation between occlusal contact area and approximate maximum bite force (*r* = 0.91, *p* < 0.001). Our findings on occlusal contact and bite force were 7.96 ± 3.55 mm^2^ and 254.3 ± 76.4 N, respectively, after 3 months of wearing implant-support prostheses.

### Measurement of efficiency of specimen mastication

The obtained glucose data varied considerably between before and immediately after implant surgery (before, 0–180.7 mg/dl, SD = 62.9 mg/dl; day 0, 23.0–258 mg/dl, SD = 73.92 mg/dl). In contrast, masticatory efficiency was not significantly different among the four periods (Wilcoxon test) (Fig. 2c). Overall, the masticatory efficiency increased by 46% immediately after surgery, stabilizing at around 40% 3 months after implant rehabilitation. This parameter was decreased in two participants at 3 months after wearing implants, one of whom also showed decreases in both occlusal contact and bite force.

We also obtained data on the healthy control group (*n* = 11), with an age similar to that of the experimental participants (age average ± SD, 65 ± 9; glucose data average ± SD, 25.5 ± 77.6). The findings for our experimental participants under all conditions were lower than those for the control group (Fig. 2c).

### Hardness assessment

**Fig. 3:**
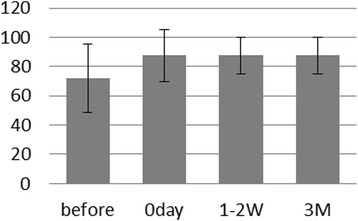
Mean and standard deviation (SD) of percentage of correct answers regarding hardness at each of the four times. The *horizontal label axis* was the time stage, and the label to the *vertical axis* was percentage of correct answers regarding hardness (%)

Hardness perception became better after implant rehabilitation, with a reduction of the error rate by 16% (Fig. 3). While five out of eight participants performed better in this test after rehabilitation, the results in the others were less clear. More detailed analysis showed that, despite wearing dentures, four participants were 100% successful in recognition of hardness before implant surgery, while four others had a 50% success rate, implying a response by chance. Noteworthy, three patients were able to chew and swallow a hard specimen immediately after implant rehabilitation.

## Discussion

Occlusal contact was significantly increased 3 months after implant rehabilitation when compared to stage one (prior to implant rehabilitation). We assumed the reason was that some participant’s occlusion was worn down because the material of provisional restoration was resin. To observe the adaptation of masticatory function after rehabilitation with an immediately loaded implant-supported prosthesis, we compared the data of four stages (before and after implant surgery) using four tests (occlusal contact, approximate maximum bite force, masticatory efficiency, and recognition of hardness threshold). The present method was simple and acceptable for use in a clinical patient setting, as the specimens had characteristics similar to typical sweets that contain glucose. In a previous study, the present method for masticatory efficiency was validated and found to be comparable to a sieve method [40].

In this study, we measured occlusal contact and maximum bite during a 3-month follow-up period in patients with implant-supported prostheses. Generally, maximum bite force was increased after 3 months, with a positive correlation to occlusal contact, in accordance with the literature [41].

We found no differences regarding the masticatory efficiency of the specimen among the different time periods, even when bite force and occlusal contact area were significantly increased. Although the present sample is small, masticatory performance seemed to be influenced by the motivation of the participants, with more improvement immediately after implant treatment. However, that is mere surmise.

### Recognition of hardness threshold

In the present study, there were no differences regarding the recognition of hardness threshold among the hardness levels at each stage. Edentulous patients with implant-supported dentures showed improved tactile discrimination ability and motor function in contrast to patients with complete dentures [42, 43] However, it is important to compare these results with those from patients with implant-supported prostheses in both jaws, lacking any kind of periodontal feedback. Trulsson [13] reported that the periodontal ligament had the highest sensitivity to changes in tooth load at low forces (below 1 N for anterior teeth and 4 N for posterior teeth). In dentate people, this may help in modulating the jaw muscles, especially when dealing with a rapid force build up, in relation to hard food.

## Conclusions

The present pilot study could not confirm an immediate rise in bite force after implant rehabilitation. Instead, improvements were mainly noted up to 3 months after surgery and rehabilitation. Furthermore, it became evident that despite gradually improved bite force in all patients, masticatory efficiency and food hardness perception did not necessarily follow the same trend. The present findings may be used to adapt oral function after implant rehabilitation by studying the combined outcome of four tests (occlusal contact, maximum bite force, masticatory efficiency, and food hardness perception). Studies with a longer follow-up time and larger sample sizes are needed to verify the present results.
